# Supplementary material for: Dicer ablation in Kiss1 neurons impairs puberty and fertility preferentially in female mice
Source: Nat Commun. 2022 Aug 9;13:4663. doi: 10.1038/s41467-022-32347-4 (PMC9363423; doi:10.1038/s41467-022-32347-4)
Supplement: Supplementary file 1 — Supplementary Information [file 41467_2022_32347_MOESM1_ESM.pdf]

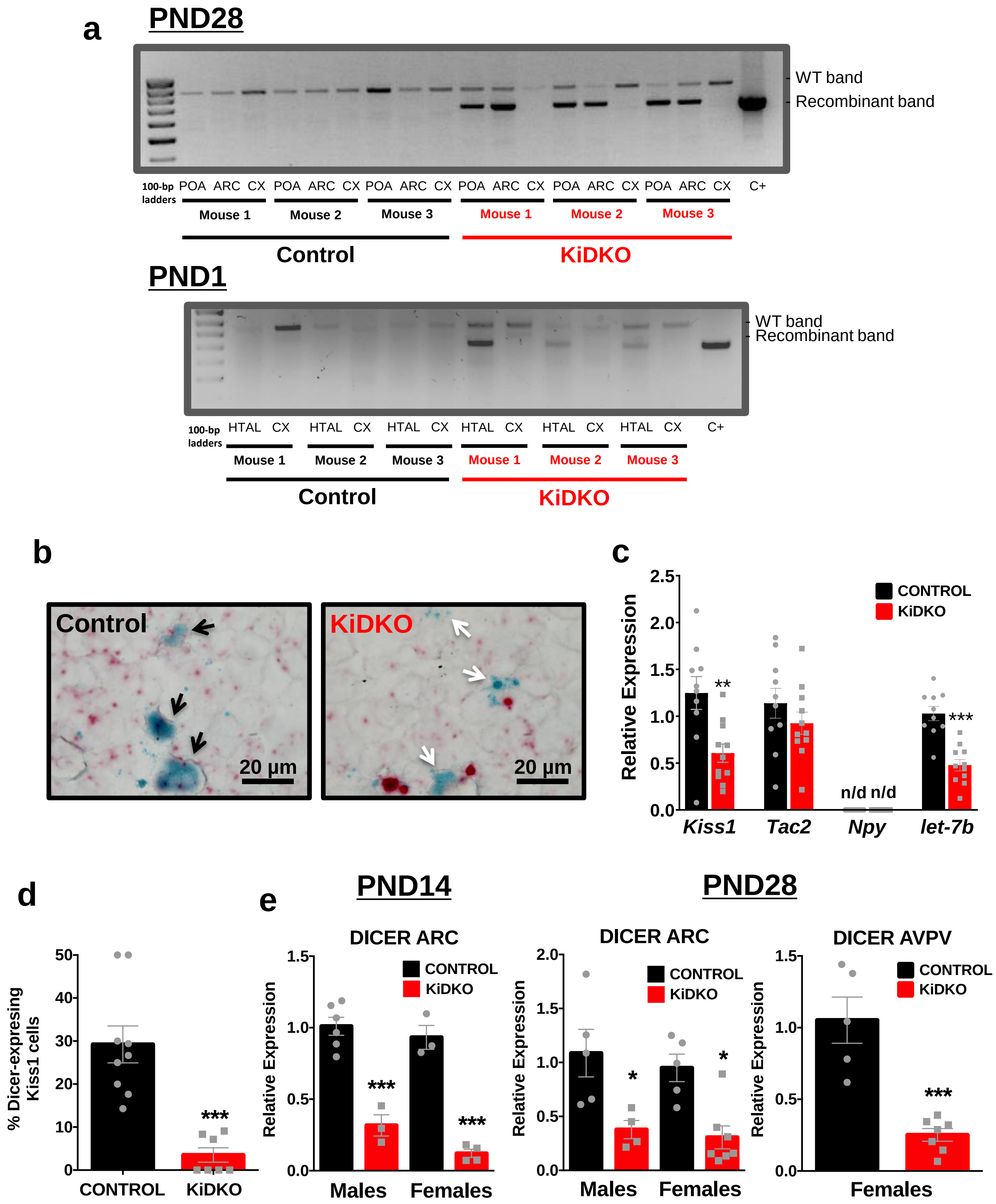

a
PND28
WT band
Recombinant band
100-bp ladders
POA ARC CX POA ARC CX POA ARC CX POA ARC CX POA ARC CX POA ARC CX C+
Mouse 1 Mouse 2 Mouse 3 Mouse 1 Mouse 2 Mouse 3
Control
KiDKO
PND1
WT band
Recombinant band
100-bp ladders
HTAL CX HTAL CX HTAL CX HTAL CX HTAL CX HTAL CX C+
Mouse 1 Mouse 2 Mouse 3 Mouse 1 Mouse 2 Mouse 3
Control
KiDKO
b
Control
KiDKO
20 µm
20 µm
c
Relative Expression
CONTROL
KiDKO
2.5
2.0
1.5
1.0
0.5
0.0
**
***
n/d n/d
Kiss1
Tac2
Npy
let-7b
d
% Dicer-expresing Kiss1 cells
50
40
30
20
10
0
***
CONTROL
KiDKO
e
PND14
PND28
DICER ARC
Relative Expression
CONTROL
KiDKO
***
***
Males
Females
DICER ARC
CONTROL
KiDKO
*
*
Males
Females
DICER AVPV
CONTROL
KiDKO
***
Females

Suppl. Figure S1. *Generation and validation of the KiDKO mouse line.* Ablation of Dicer in Kiss1 neurons was achieved in vivo using a Cre-LoxP approach, by crossing a Kiss1-Cre mouse line with a Dicer$^{loxP/loxP}$ mouse line. Panel (**a**) illustrates PCR detection of the recombinant event leading to Dicer allele deletion in the preoptic area (POA) and arcuate nucleus (ARC), areas with prominent Kiss1 neuronal populations, but not in the cortex (as tissue devoid of Kiss1 cells), in 28 day-old mice (PND28). In addition, recombination was assessed also in the hypothalamus at PND1. An amplicon of expected size, denoting effective recombination, was detected in the ARC, POA and whole hypothalamus of KiDKO mice, whose identity was confirmed by direct sequencing. In contrast, no amplification was observed in the cortex of KiDKO mice, neither in the POA or cortex of control mice. Three individuals per genotype were analyzed. In addition, in panel **b**, results of dual labeling, using BaseScope^{TM} in situ hybridization in OVX females at PND54, are presented. Note that, while in controls, Kiss1 cells (labelled in green) are shown to co-express Dicer exon 23 (labelled in red; see *black arrows*), in KiDKO mouse hypothalamus, such co-expression drops to virtually negligible levels, despite detectable kisspeptin content. Scales In panel (**c**), TaqMan qPCR analyses in FACS isolated Kiss1 neurons from peripubertal (PND28) KiDKO-YFP mice are shown. Despite preserved expression of *Tac2* gene in ARC Kiss1 neurons, *Kiss1* mRNA levels were partially decreased and expression of an abundant miRNA, namely let-7b, was markedly suppressed in KiDKO mice, the latter attesting for effective ablation of the miRNA synthesizing enzyme, Dicer, in our mouse line. In addition, *Npy* mRNA was not detected in qPCR assays, demonstrating the absence of contamination with other abundant ARC cells in FACS isolated Kiss1 neuronal samples. Group sizes: n= 10 control; n=11 KiDKO. On the other hand, single-cell analysis of FACS isolated Kiss1 neurons demonstrated a dramatic reduction in the percentage of Kiss1 neurons expressing Dicer in KiDKO mice (**d**). Group sizes: n= 9 control; n=7 KiDKO animals. In the same context, *Dicer* expression was significantly reduced in ARC Kiss1 neurons isolated by FACS from infantile (PND14) and peripubertal (PND28) KiDKO male and female mice, and in AVPV Kiss1 neurons from peripubertal females (**e**). Group sizes: PND14 (n= 6 control; n=3 KiDKO males and n= 3 control; n=4 KiDKO females); PND28 (n= 5 control; n=4 KiDKO males and n= 5 control; n=7 KiDKO females). The values are represented as the mean ± SEM. * $P < 0.05$; **$P < 0.01$; ***$P < 0.001$ vs. corresponding control groups. nd= not detectable.

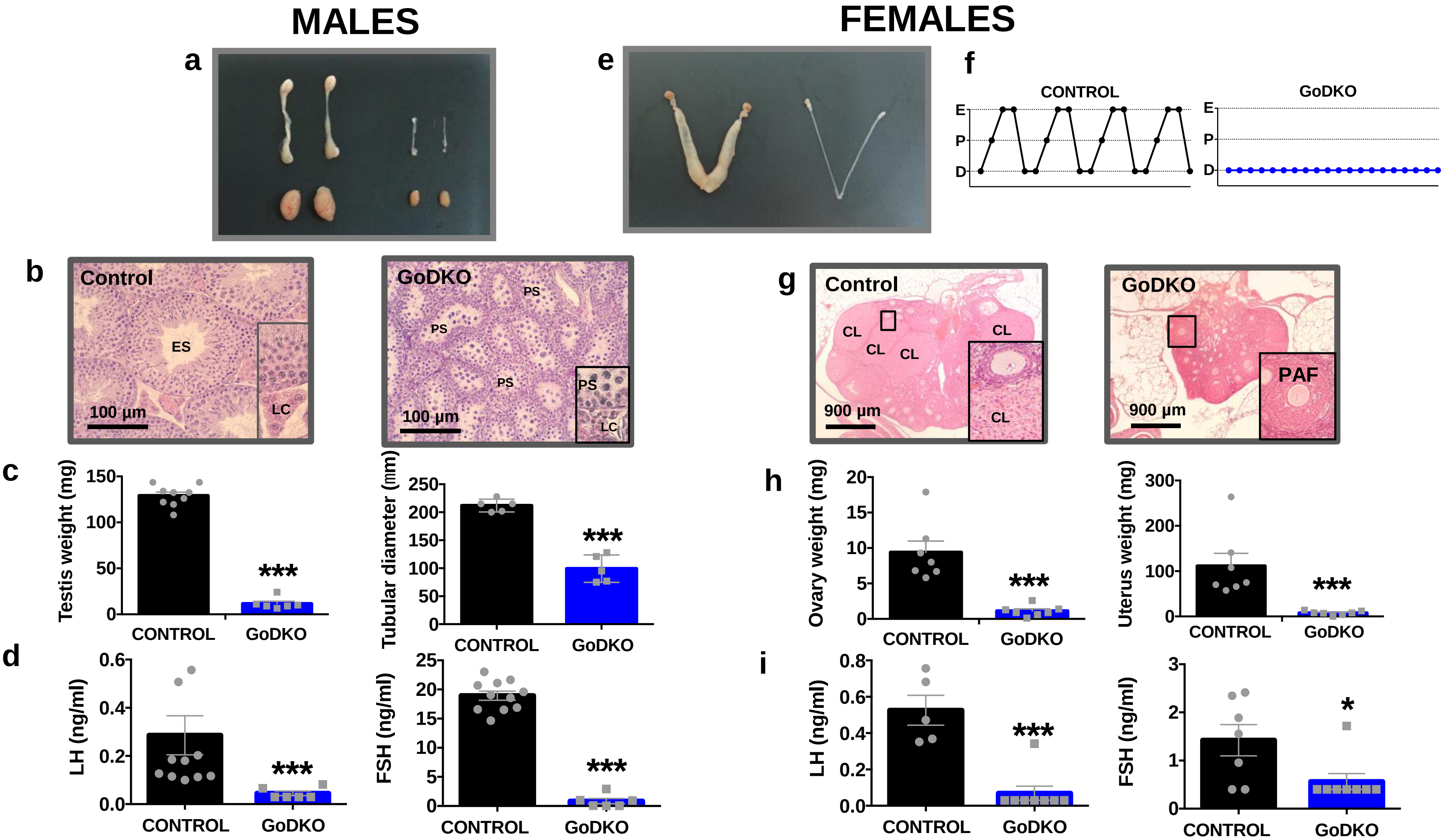

Suppl. Figure S2. *Profound hypogonadotropic hypogonadism in adult GoDKO mice*. Representative images of the gonads (testes, ovaries) and sex organs (epididymis, uterus) for adult control and GoDKO mice of both sexes are presented in (**a**; males) and (**e**; females). For the latter, individual profiles of ovarian cyclicity, monitored by vaginal cytology, are also shown in (**g**). In addition, representative histological sections of the testis (**b**) and ovary (**g**) are presented for control and GoDKO mice. Control mice showed full spermatogenesis, denoted by the presence abundant elongated spermatids (ES), and prominent Leydig cells (LC) in the interstitium. In contrast, KO mice showed atrophic seminiferous tubules with spermatogenic arrest at the primary spermatocyte (PS) level, as well as poorly differentiated Leydig cells. In the case of the ovary, while control females showed cycling ovaries with abundant growing follicles and corpora lutea (CL), in GoDKO females large antral follicles and corpora lutea were not found in KO mice, while preantral (PAF; see *inset*) or early antral follicles were the most advanced healthy growing follicles. In addition, testicular weight and tubular diameter in males (**c**; control n=9, GoDKO n=6 for testis weigh and control n=5, GoDKO n=5 for tubular diameter), and ovarian and uterus weight in females (**h**; control n=7, GoDKO n=7) are presented for control and GoDKO mice, for which serum LH and FSH levels are also shown (**d**; control n=11; GoDKO n=6 for both LH and FSH, **I**; control n=5 for LH and control n=7 for FSH; GoDKO n=8 for both LH and FSH). The values are represented as the mean ± SEM. * $P < 0.05$; ***$P < 0.001$ vs. corresponding control groups.

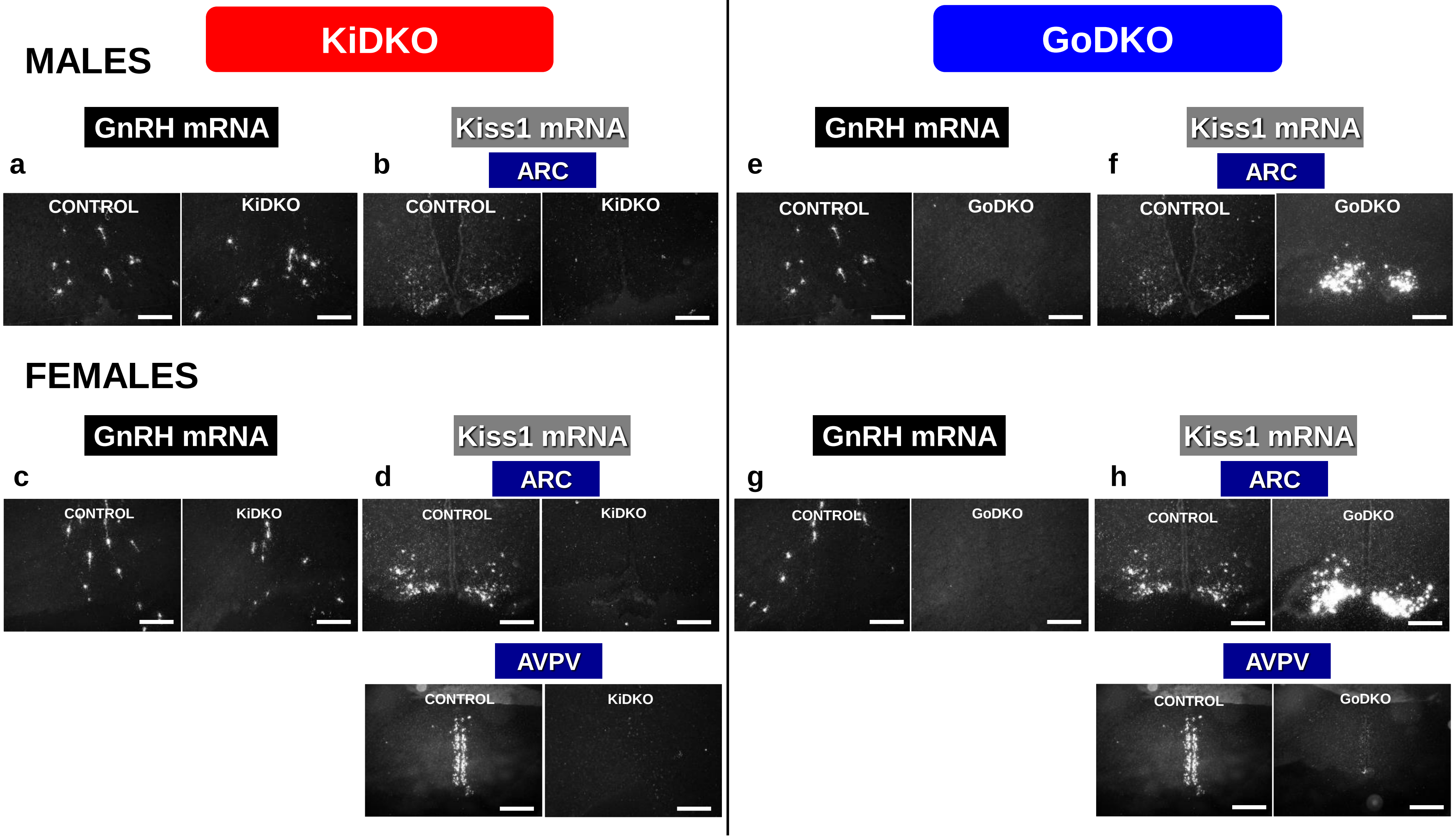

Suppl. Figure S3. *Changes in hypothalamic Kiss1 and GnRH expression in KiDKO and GoDKO mice.* Expression of *Kiss1* and *GnRH* mRNAs in specific hypothalamic areas (*Kiss1*: ARC and AVPV; *GnRH*: POA) of the two mouse lines was assessed by in situ hybridization (ISH). POA *GnRH* expression was fully preserved in male (**a**) and female (**c**) adult KiDKO mice, whereas in adult GoDKO animals it was totally absent (**e**, males; **g**, females). In turn, Kiss1 mRNA levels were undetectable in the ARC of male (**b**) and female (**d**) KiDKO mice, as well as in the AVPV of female KiDKO mice (**d**). In contrast, enhanced *Kiss1* expression was detected in the ARC of male (**f**) and female (**h**) GoDKO mice, possibly due to the lift of the sex steroid negative feedback caused by hypogonadism in this line. Such decrease in ovarian steroids is likely causative for the substantial suppression of *Kiss1* mRNA levels seen in the AVPV of female GoDKO mice (**h**). Scale bars correspond to 200 µm.

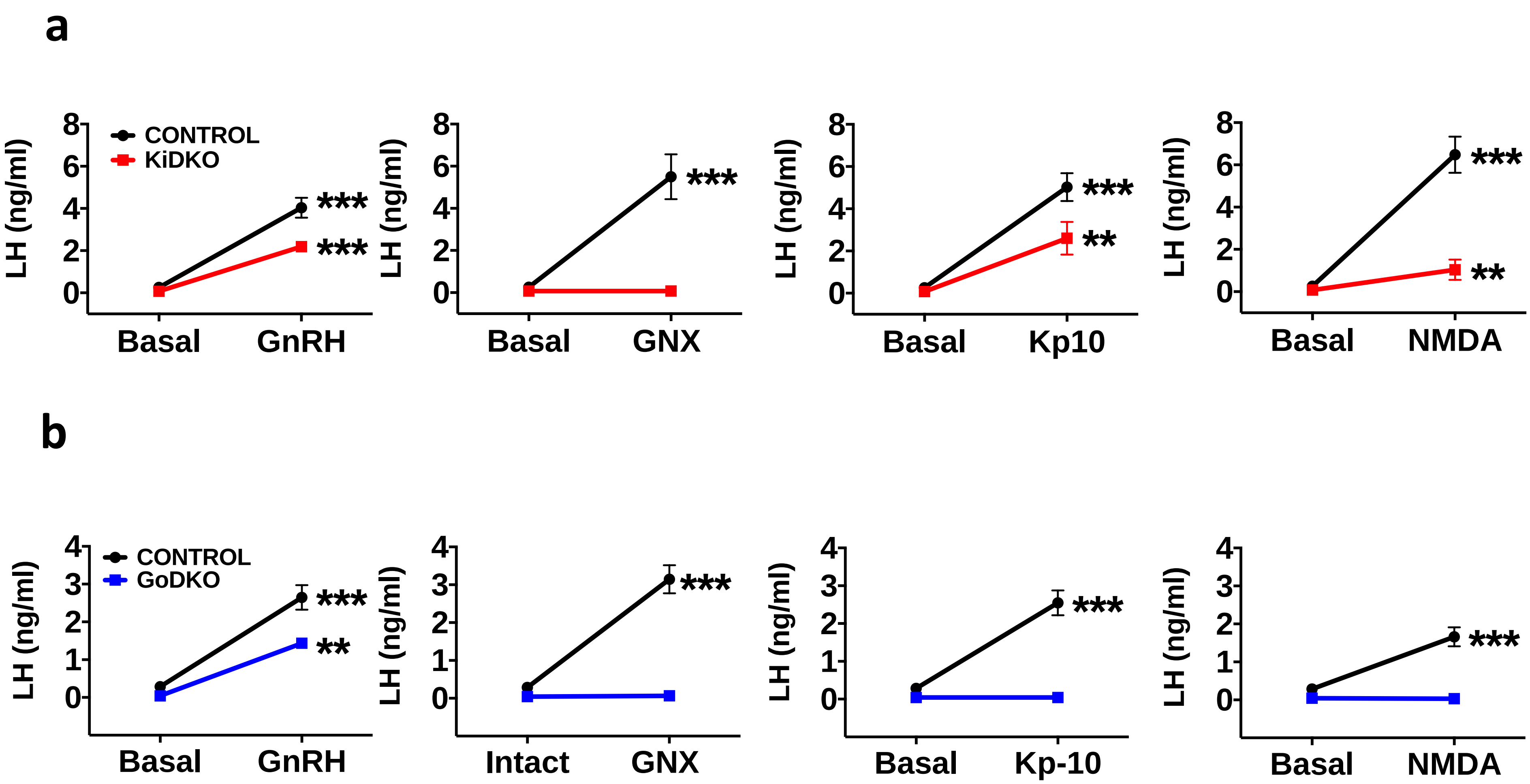

Suppl. Figure S4. *LH responses to different neuroendocrine stimuli in adult KiDKO and GoDKO* male *mice.* In upper panels (**a**), LH levels in adult KiDKO mice, at basal conditions and after administration of a single bolus of GnRH (0.25 μg, ip), Kp-10 (1 nmol, icv) or NMDA (1 nmol, icv) are presented. In addition, changes in serum LH levels, 2-weeks after gonadectomy (GNX), in KiDKO mice are shown. Similar analyses were conducted in adult GoDKO mice, and data are presented in the lower panels (**b**). Group sizes, for the KiDKO line: n=6 control in basal conditions and n=7 control after injection; n=5 KiDKO in basal conditions and n=8 KiDKO after injection; for the GoDKO line: n=11 control in basal conditions and n=10 control after injection; n=6 GoDKO in basal conditions and n=5 GoDKO after injection. The values are represented as the mean ± SEM. **P <0.01; ***P < 0.001 vs. corresponding basal groups.

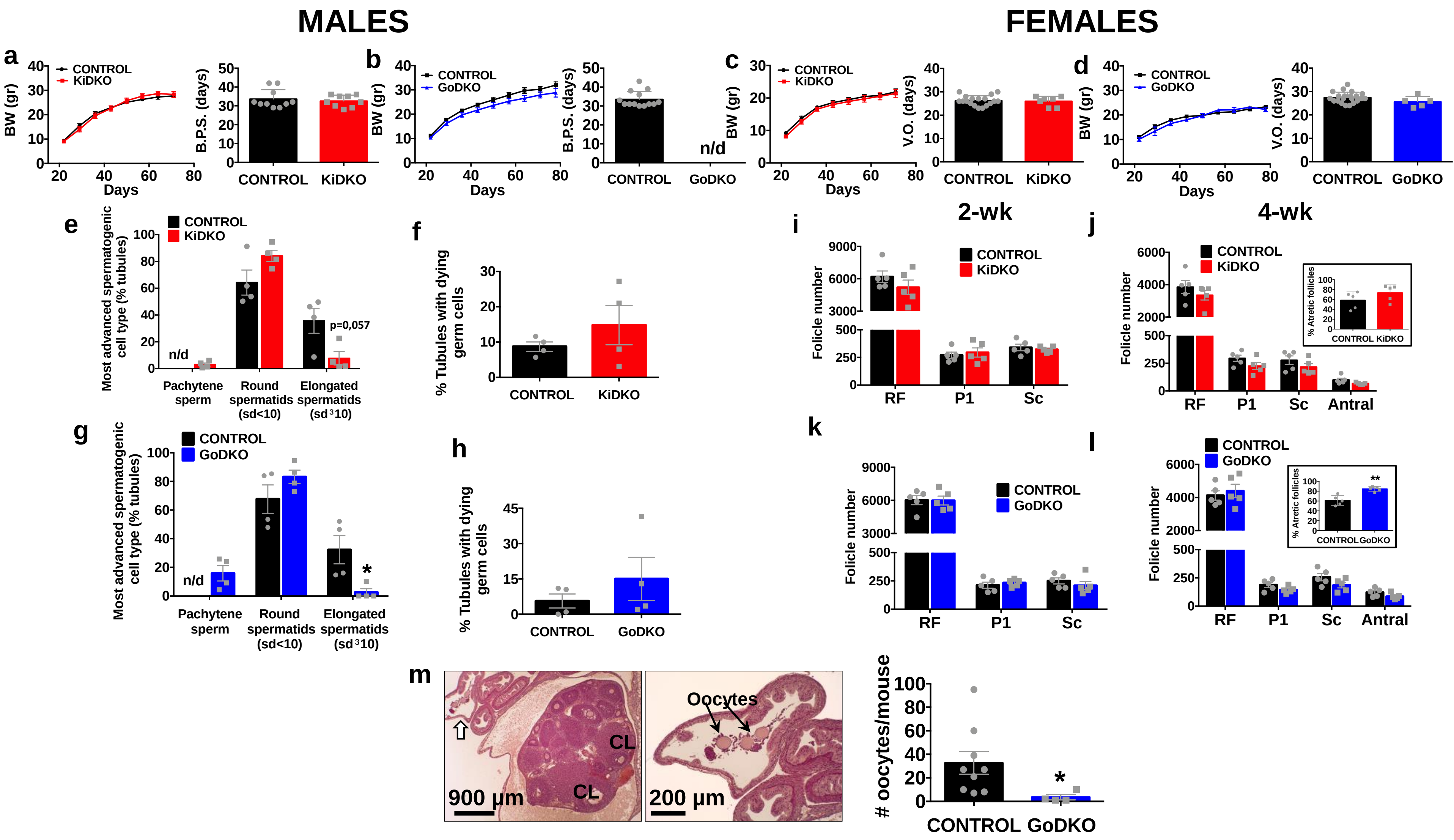

Suppl. Figure S5. *Reproductive indices in KiDKO and GoDKO mice at puberty and adulthood*. Data from the two models, KiDKO (Kiss1-specific Dicer KO) and GoDKO (GnRH-specific Dicer KO), are presented. Postnatal body weight (BW) gain and balano-preputial separation (BPS, as external marker of puberty) are presented for male KiDKO (**a**; control n=10; KiDKO n=10) and GoDKO (**b**; control n=11, GoDKO n=8) mice, and their corresponding controls. In addition, quantitative parameters of spermato-genesis, including the most advanced spermatogenic cell type and % of tubules with dying cells, are presented for KiDKO (**e**, **f**; control n=4; KiDKO n=4) and GoDKO (**g**, **h**; control n=4; GoDKO n=4) males. In the case of females, postnatal BW gain and vaginal opening (VO, as external marker of puberty) are presented for KiDKO (**c**; BW: control n=5, KiDKO n=7; and VO: control n=18, KiDKO n=7) and GoDKO (**d**; control n=21; GoDKO n=5) mice, and their corresponding controls. In addition, quantitative parameters of folliculogenesis, including follicle numbers at 2-wk and 4-wk of postnatal life, as well as the % of atretic follicles (see *insets*), are shown for KiDKO (**i**, **j**; control n=5; KiDKO n=5) and GoDKO (**k**, **l**; control n=5; GoDKO n=5) females. Finally, in panel (**m**), representative histological images of ovaries of GoDKO female mice after ovulation induction by standard gonadotropin priming are shown, with appearance of newly formed corpora lutea (CL) and released oocytes in the uterine tubes (denoted by *arrows*). Quantification of the #oocytes observed in control and GoDKO females after gonadotropin priming is also displayed (control n=9; GoDKO n=4). The values are represented as the mean ± SEM. * P < 0.05; **P < 0.01 vs. corresponding control groups. nd= not detectable.

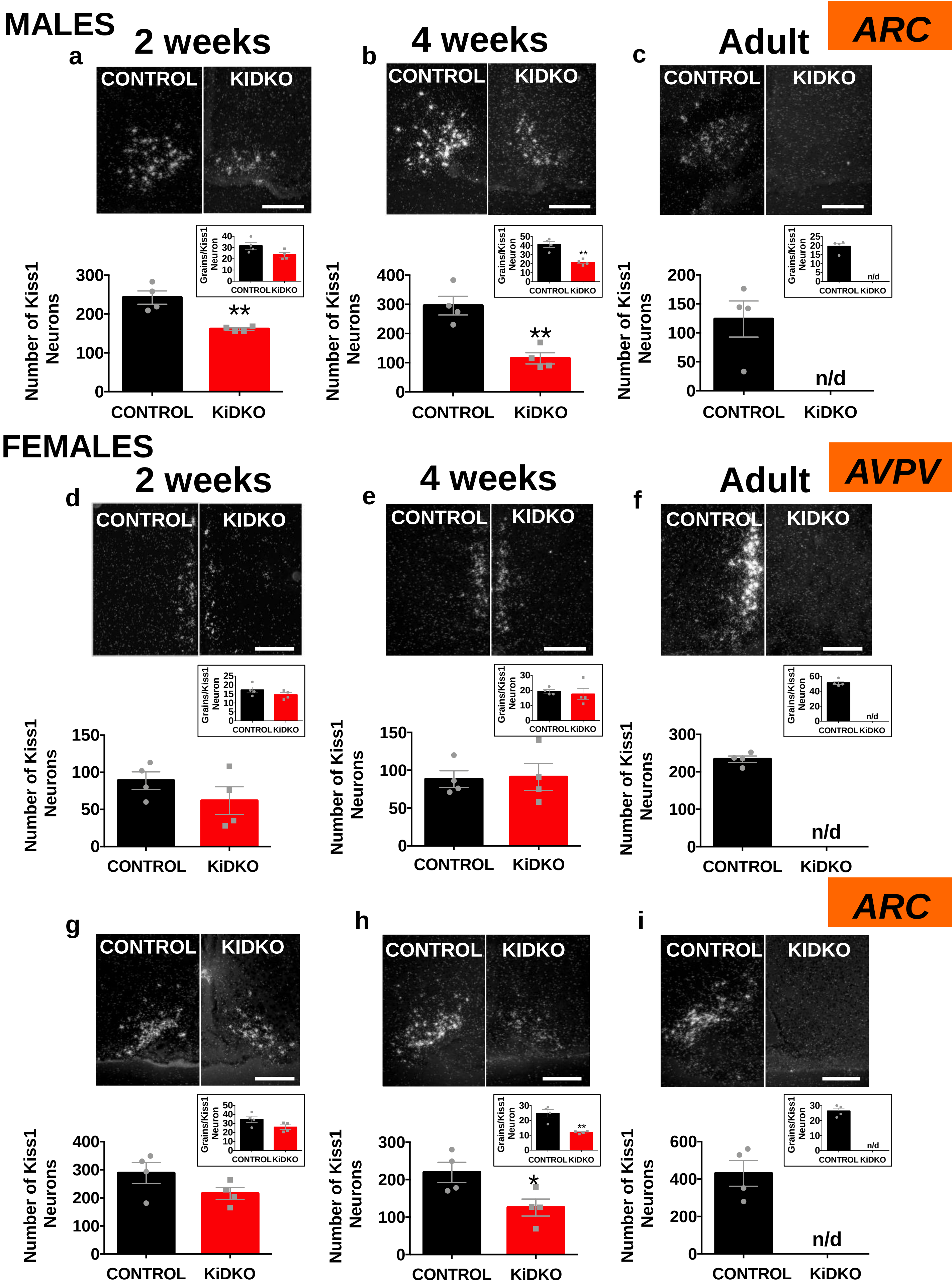
MALES
ARC
2 weeks
4 weeks
Adult
a
b
c
CONTROL
KIDKO
Number of Kiss1 Neurons
Grains/Kiss1 Neuron
CONTROL
KiDKO
**
n/d
FEMALES
AVPV
d
e
f
ARC
g
h
i
*

Suppl. Figure S6. *Hypothalamic Kiss1 expression in KiDKO mice along postnatal maturation*. Expression of *Kiss1* mRNA at the ARC and AVPV was assessed by in situ hybridization (ISH). This procedure allowed counting of numbers of *Kiss1*-expressing cells, as well as the grain density per cell (as proxy marker of individual expression). Representative images and quantitative data on #*Kiss1*-expressing neurons and *Kiss1* grain density per cell, are shown in the ARC of males (**a**-**c**), and the AVPV (**d**-**f**) and ARC (**g**-**i**) of females, of control and KiDKO genotypes. Data were collected at three postnatal ages: 2-weeks (corresponding to mini-puberty); 4-weeks (corresponding to early pubertal transition); and adulthood (4-months). Group sizes: n=4 control males; n=4 KiDKO males; n=4 control females; n=4 KiDKO females. The values are represented as the mean ± SEM. * $P < 0.05$; **$P < 0.01$ vs. corresponding control groups. Scale bars correspond to 200 µm.

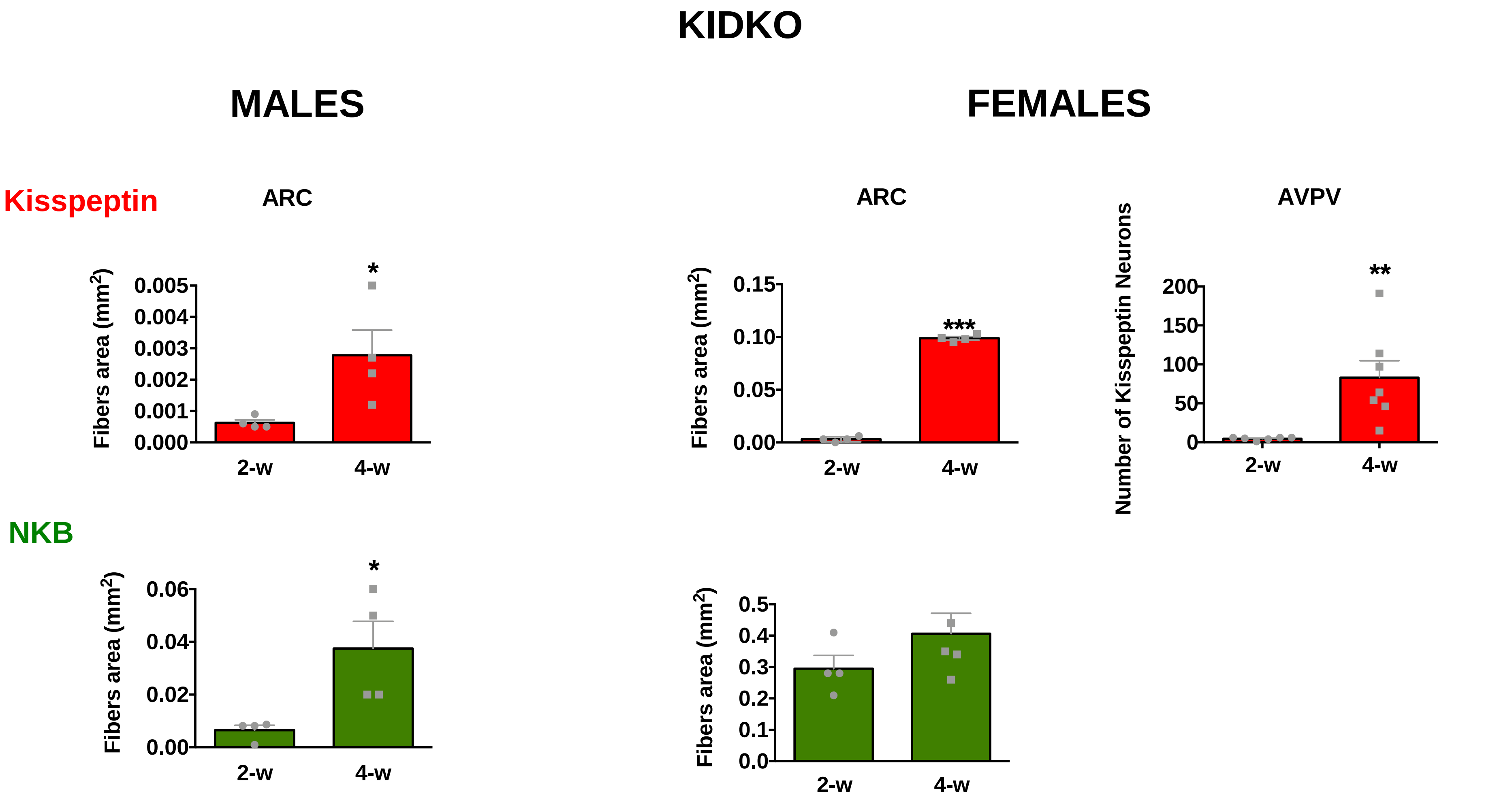

Suppl. Figure S7. *Changes in kisspeptin and NKB levels in KiDKO mice during the pubertal transition*. Quantification of changes in kisspeptin- (in red) and NKB- (in green) immunoreactivity (IR) in the ARC of male and female KiDKO mice, and of kisspeptin-IR in the AVPV of female KiDKO mice, between 2-wk and 4-wk of postnatal age. Group sizes for ARC densitometry: n= 4 control males; n = 4 KiDKO males; n = 4 control females; n= 4 KiDKO females. Of note, for quantification of kisspeptin cell number in the AVPV, a higher number of animals (n=6 for 2- and n=7 for 4-weeks), was included due to the greater variability. The values are represented as the mean ± SEM. * $P < 0.05$; **$P < 0.01$; ***$P < 0.001$ vs. corresponding 2-wk group.

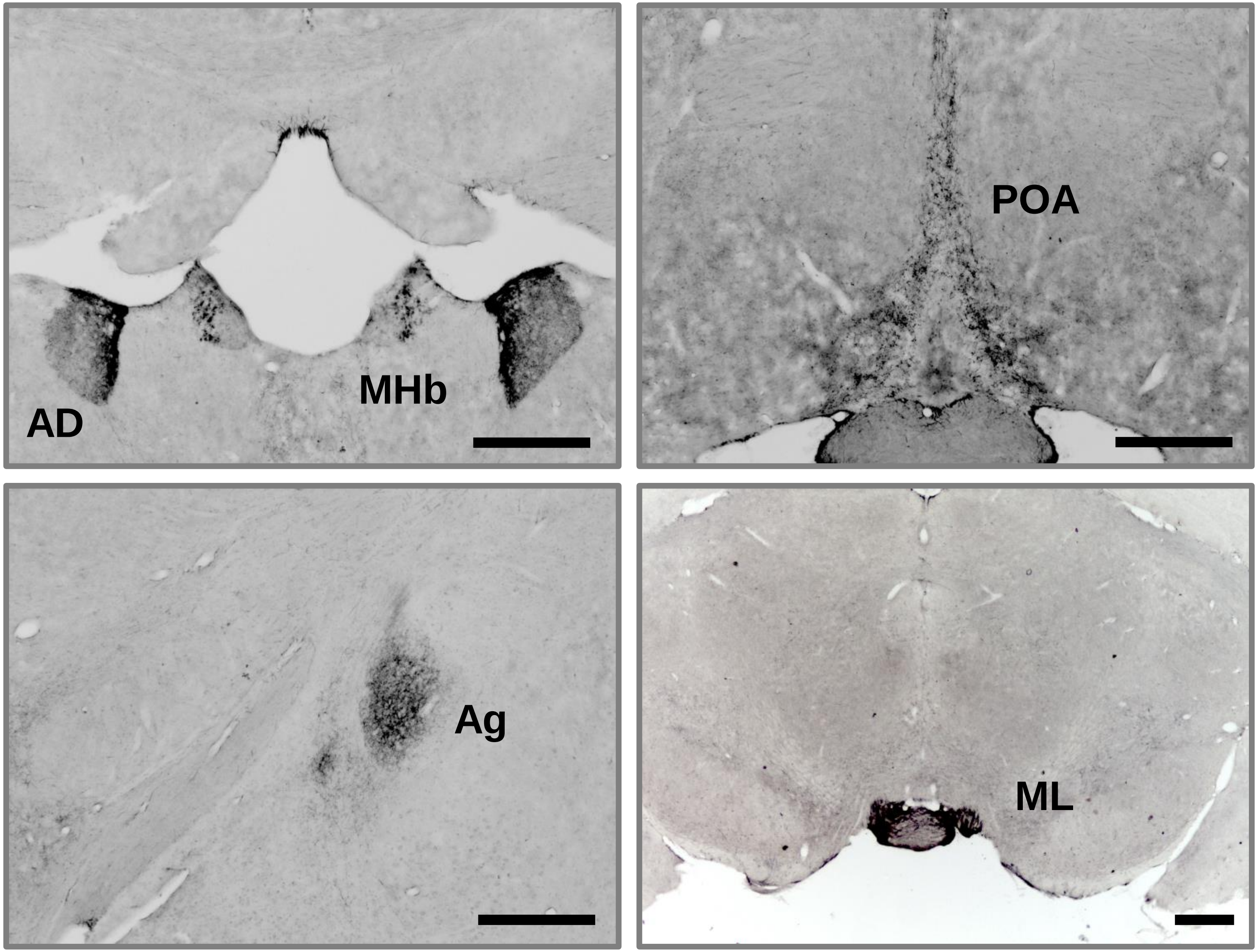

MHb: Medial habenular nucleus; **AD**: Anterodorsal nucleus; **POA**: Preoptic area;
Ag: Amygdala; **ML**: Medial Mammillary nucleus

Suppl. Figure S8. *Persistent NKB expression in other brain areas of KiDKO mice.* Representative images of NKB immunoreactivity in various brain areas of KiDKO mice, where NKB expression has been previously reported, including the medial habenular nucleus (MHB), the antero-dorsal nucleus (AD), the preoptic area (POA), and the medial mammillary nucleus (ML). Scale bars correspond to 200 µm.

Suppl. Table S1. Sequences of primers for in situ hybridization (ISH)

Riboprobe	*GenBank Accession number*	*Primers*	*Sequence*	*Spaning Region*
**Kiss1**	***NM_178260.3***	Externals	Kiss1 forward 5'-ATG ATC TCA ATG GCT TCT TGG-3'	39…419
			Kiss1 reverse 5'-TCA GCC CCG TGC TGC CCG CGC-3'	
		Internals	T3-Kiss1 forward 5'-CAG AGA TGC AAT TAA CCC TCA CTA AAG GGA GAG TGA AGC CTG GAT CCA CAG GC-3'	111...397
			T7-Kiss1 reverse 5'-CCA AGC CTT CTA ATA CGA CTC ACT ATA GGG AGA GCC TGC CTC CTG CCG TAG CGC-3'	
GnRH	***NM_008145.2***	Externals	GnRH forward 5'-GTT GAC TGT GTG TTT GGA AGG C-3'	137...371
			GnRH reverse 5'-CTT CTT CTG CCC AGC TTC CTC-3'	
		Internals	T3-GnRH forward 5'-CAG AGA TGC AAT TAA CCC TCA CTA AAG GGA GAA GCA CTG GTC CTA TGG GTT GCG-3'	170…344
			T7-GnRH reverse 5'-CCA AGC CTT CTA ATA CGA CTC ACT ATA GGG AGA CAG ACG TTC CAG AGC TCC TCG-3'	
