# Supplementary material for: Dicer ablation in Kiss1 neurons impairs puberty and fertility preferentially in female mice
Source: Nat Commun. 2022 Aug 9;13:4663. doi: 10.1038/s41467-022-32347-4 (PMC9363423; doi:10.1038/s41467-022-32347-4)
Supplement: Supplementary file 2 — Reporting Summary [file 41467_2022_32347_MOESM2_ESM.pdf]

nature research

Corresponding author(s): Juan Roa and Manuel Tena-Sempere

Last updated by author(s): Jun 3, 2022

Reporting Summary

Nature Research wishes to improve the reproducibility of the work that we publish. This form provides structure for consistency and transparency in reporting. For further information on Nature Research policies, see our Editorial Policies and the Editorial Policy Checklist.

Statistics

For all statistical analyses, confirm that the following items are present in the figure legend, table legend, main text, or Methods section.

n/a	Confirmed	
☐	☒	The exact sample size (*n*) for each experimental group/condition, given as a discrete number and unit of measurement
☐	☒	A statement on whether measurements were taken from distinct samples or whether the same sample was measured repeatedly
☐	☒	The statistical test(s) used AND whether they are one- or two-sided<br>*Only common tests should be described solely by name; describe more complex techniques in the Methods section.*
☒	☐	A description of all covariates tested
☐	☒	A description of any assumptions or corrections, such as tests of normality and adjustment for multiple comparisons
☐	☒	A full description of the statistical parameters including central tendency (e.g. means) or other basic estimates (e.g. regression coefficient) AND variation (e.g. standard deviation) or associated estimates of uncertainty (e.g. confidence intervals)
☐	☒	For null hypothesis testing, the test statistic (e.g. F, t, r) with confidence intervals, effect sizes, degrees of freedom and P value noted<br>*Give P values as exact values whenever suitable.*
☒	☐	For Bayesian analysis, information on the choice of priors and Markov chain Monte Carlo settings
☒	☐	For hierarchical and complex designs, identification of the appropriate level for tests and full reporting of outcomes
☒	☐	Estimates of effect sizes (e.g. Cohen's d, Pearson's r), indicating how they were calculated

Our web collection on statistics for biologists contains articles on many of the points above.

Software and code

Policy information about availability of computer code

Data collection	Image adquisition and counting on gonadal histologicals sections were done using an Elipse 400 microscope and provided propietary software for image adquisition.<br>Image adquisition and counting on brain histological sections were done using an Leica DM2500 microscope and provided propietary software for image adquisition<br>qPCR data were obtained using a CFX96 Touch Real-Time PCR Detection System (Bio-Rad) and provided propietary software.
Data analysis	The area containing Kisspeptin and pro-NKB immuno-reactivity was quantified by densitometry using Image J 1.53k (NIH).<br>Kiss1 and GnRH ISH sections were quantified using custom-designed software, enabled to count the total number of cells (grain clusters) as well as the number of silver grains/cell.<br>Statistical analysis were done using Prism GraphPad 9.0 software.

For manuscripts utilizing custom algorithms or software that are central to the research but not yet described in published literature, software must be made available to editors and reviewers. We strongly encourage code deposition in a community repository (e.g. GitHub). See the Nature Research guidelines for submitting code & software for further information.

Data

Policy information about availability of data

All manuscripts must include a data availability statement. This statement should provide the following information, where applicable:

- Accession codes, unique identifiers, or web links for publicly available datasets
- A list of figures that have associated raw data
- A description of any restrictions on data availability

A data availability statement is included in the MS. All source data (and statistical analyses) are also provided, as source data file, in a single Excel document

Field-specific reporting

Please select the one below that is the best fit for your research. If you are not sure, read the appropriate sections before making your selection.

[x] Life sciences [ ] Behavioural & social sciences [ ] Ecological, evolutionary & environmental sciences

For a reference copy of the document with all sections, see nature.com/documents/nr-reporting-summary-flat.pdf

Life sciences study design

All studies must disclose on these points even when the disclosure is negative.

Sample size	As general rule, sample sizes were selected based on our previous experience with studies addressing neuroendocrine regulation of puberty and the reproductive axis, assisted by power analyses performed using values of standard deviation that we usually obtain when measuring parameters analogous to those examined in this study. Based on those calculations, minimal group sizes of n= 6 animal per group were established, unless otherwise indicated due to operational reasons, as analyses using these sample size should provide at least 80% power to detect effect sizes using the tests indicated above, with a significance level of 0.05.
Data exclusions	No data were excluded from the analyses.
Replication	As a general rule, experiments were performed with the calculated sample size to determine statistical differences and no replication of individual experiments were performed. However, some experiments based on different tecniques confirmed the same results by different ways. For example:<br>1. To validate the specificity of conditional ablation of Dicer in Kiss1 cells in the KiDKO line, three different approaches were used: PCR detection of the recombinated Dicer-flox allele in hypothalamic regions containing Kiss1 neurons, BaseScope ISH of Kiss1 and Dicer mRNA and qPCR detection of Dicer mRNA and the mature miRNA, let-7b-5p, in FACS-isolated Kiss1 neurons. (Fig S1)<br>2. The drop of Kiss1 mRNA in the ARC of peripubertal KiDKO mice was detected both by ISH of Kiss1 mRNA and qPCR detection of Kiss1 mRNA in FACS-isolated cells. Also, these results were confirmed indirectly in the IHC analysis of Cre-GFP expression, which is dependent on Kiss1 promoter activity and showed reduced number of GFP-positive cells in the ARC of prepubertal KiDKO mice. (Fig S6b,h; Fig S1c and Fig 3).<br>3. The drop of basal LH on adult male KiDKO mice and the maintenance of basal LH levels of females KiDKO mice were confirmed on single-point determinations of LH by RIA on serum samples and from tail-tip bleeding blood samples determination of LH by ELISA taken in pulsatility experiments (Fig.1d,e,j,k).
Randomization	While phenotypic and hormonal analyses were applied to all available samples, more complex molecular/histological analyses in these experiments were conducted in a representative subset of randomly assigned samples from each group.
Blinding	As general procedure, the investigators directly performing the experimentation involving physiological/molecular determinations were not blinded to the group allocation, but primary data analyses conducted by senior authors were conducted independently to avoid any potential bias.

Reporting for specific materials, systems and methods

We require information from authors about some types of materials, experimental systems and methods used in many studies. Here, indicate whether each material, system or method listed is relevant to your study. If you are not sure if a list item applies to your research, read the appropriate section before selecting a response.

Materials & experimental systems

n/a	Involved in the study
[ ]	[x] Antibodies
[x]	[ ] Eukaryotic cell lines
[x]	[ ] Palaeontology and archaeology
[ ]	[x] Animals and other organisms
[x]	[ ] Human research participants
[x]	[ ] Clinical data
[x]	[ ] Dual use research of concern

Methods

n/a	Involved in the study
[x]	[ ] ChIP-seq
[ ]	[x] Flow cytometry
[x]	[ ] MRI-based neuroimaging

Antibodies

Antibodies used	Primary antibodies:<br>- RIA kits for LH and FSH measurements from the National Institutes of Health (Dr. A. F. Parlow, National Hormone and Peptide Program, Torrance, CA).<br>- Anti-bovine LHβ subunit, 518B7 (L. Sibley; University of California, UC Davis). RRID:AB_2756886.<br>- Rabbit LH antiserum, AFP240580Rb provided by NIDDK-NHPP. RRID:AB_2784499.<br>- Rabbit anti-rat/mouse kisspeptin antibody, AC#566, from Dr Alain Caraty, PRC-INRA, 37380 Nouzilly, France. RRID:AB_2314707.<br>- Rabbit anti-proNKB, IS39, from Dr Philippe Ciofi, INSERM U 1215, 33077 Bordeaux, France. RRID:AB_2819032.<br>- Chicken anti-GFP antibody. Abcam; Cat. Ab13970.<br><br>Secondary antibodies:<br>- Biotinylated Donkey anti-rabbit. Code 711-066-152. Jackson ImmunoResearch Europe Ltd., UK.<br>- Biotinylated Donkey anti-chicken. Code 711-066-152. Jackson ImmunoResearch Europe Ltd., UK.
Validation	- RIA kits for LH and FSH measurements. PMID: 7399110. PMID: 5725274.<br>- Anti-bovine LHβ subunit, 518B7. PMID: 3507889. PMID: 24092638.<br>- Rabbit LH antiserum, AFP240580Rb. PMID: 24092638.<br>- Rabbit anti-rat/mouse kisspeptin antibody, AC#566. PMID: 19515163.<br>- Rabbit anti-proNKB, IS39. PMID: 16809008.<br>- Chicken anti-GFP antibody. See manufacter datasheet, where it is stated that the antibody is validated for immunohistochemistry in GFP-transfected NIH3T3 cells.

Animals and other organisms

Policy information about studies involving animals; ARRIVE guidelines recommended for reporting animal research

Laboratory animals	Genetically modified C57Bl6 mice were used, the strains are detailed below. Mice from both sexes were used at PND1, PND14, PND28, PND54 and adults >2 months-old.<br>- Kiss1-Cre mice: JAX Strain #017701<br>- GnRH-Cre mice: JAX Strain #021207<br>- Dicer-flox mice: JAX Strain #006366<br>- (ROSA26)-YFP Cre-dependent reporter mice: JAX Strain #006148
Wild animals	This study did not involve wild animals.
Field-collected samples	This study did not involve samples collected from the field.
Ethics oversight	The experiments and animal protocols included in this study were approved by the Ethical Committee of the University of Cordoba; all experiments were conducted in accordance with European Union (EU) normative for the use and care of experimental animals (EU Directive 2010/63/UE, September 2010).

Note that full information on the approval of the study protocol must also be provided in the manuscript.

Flow Cytometry

Plots

Confirm that:

- [x] The axis labels state the marker and fluorochrome used (e.g. CD4-FITC).
- [x] The axis scales are clearly visible. Include numbers along axes only for bottom left plot of group (a 'group' is an analysis of identical markers).
- [x] All plots are contour plots with outliers or pseudocolor plots.
- [x] A numerical value for number of cells or percentage (with statistics) is provided.

Methodology

Sample preparation	NOTE: PLOTS SHOWING THE STRATEGY FOR ISOLATION OF KISS1 NEURONS IN KiDKO-YFP MICE ARE AVAILABLE IN OUR RECENTLY PUBLISHED ARTICLE: Heras V. et al. PLoS Biol. 2019 Nov 7;17(11):e3000532. PMID: 31697675<br>To isolate Kiss1 neurons, a triple transgenic mouse line expressing (ROSA26-YFP) Cre-dependently in the KiDKO background was used. For experiments, Kiss1Cre/-::DicerloxP/loxP::YFPloxP/-, namely KiDKO-YFP mice, and Kiss1Cre/-::DicerloxP/-::YFPloxP/-, namely control-YFP mice, were used. Kiss1 neurons populations from the AVPV and ARC were isolated separately from hypothalamic tissue blocks containing the POA and the medio-basal hypothalamus (MBH), respectively. After microdissection, POA and MBH tissue blocks were enzymatically dissociated using a Papain Dissociation System (Worthington, Lakewood, NJ) to obtain single-cell suspensions.
Instrument	FACS was performed on these suspensions with a FACS Aria III Sorter (BD Biosciences).
Software	Data were collected using FACSDiva 8.0 software (BD Biosciences). Data were analyzed using the Kaluza 2.0 software (Beckman Coulter).
Cell population abundance	A total of 229 ± 37 AVPV and 574 ± 43 ARC YFP-positive neurons were collected per animal on 10 µL of lysis buffer (0.1% Triton X-100, and 0.4 unit/µl RNAsin, Promega). For the individual isolation of ARC Kiss1 neurons, 17 ± 1 individual neurons per animal were individually sorted on 96-wells plates containing 10 µL of lysis buffer per well.
Gating strategy	First, cellular debris was excluded by gating cells for forward scatter (FSC) area and side scatter (SSC) area, and later, aggregated cells were excluded by selecting singlets by plotting FSC area vs FSC height. Finally, the sort decision was based on measurements of YFP fluorescence (excitation: 488 nm; detection: YFP band-pass 530/30 nm, autofluorescence bandpass 780/60 nm) by comparison with cell suspensions from cortex, which does not harbor YFP-positive neurons.

☐ Tick this box to confirm that a figure exemplifying the gating strategy is provided in the Supplementary Information.
